# 

**Published:** 1894-04

**Authors:** 


### TABLE OF CONTENTS.

ORIGINAL COMMUNICATIONS. PAGE

Scallop Wire Mechanism for regulating Teeth. By J. N. FARRAR, M.D., D.D.S., New York City . . . . . . . . . . 209

Alloys as Nostrums and as Subjects for Investigation. By DR. J. MORGAN HOWE, New York . . . . . . . . . . 211

Pyorrhœa Alveolaris. By C. N. PEIRCE, D.D.S., Philadelphia . . . . . . 217

Pyorrhœa Alveolaris—The Other View of it. By GEORGE S. ALLAN, D.D.S., New York . . . . . . . . . . 219

Interesting and Rare Case. By E. S. TALBOT, D.D.S., Chicago . . . . . 230

Some Observations upon Pyorrhœa Alveolaris. Review of Professor Peirce's Paper. By R. OTTOLENGUI, M.D.S., New York . . . . . . . . . . 230

Review of Professor C. N. Peirce's Recent Conclusions in regard to Pyorrhœa Alveolaris. By JAMES TRUMAN, D.D.S . . . . . . . . . . 236

Review of Professor Peirce's Paper, "Pyorrhœa Alveolaris." By DR. R. R. ANDREWS, Boston, Mass. . . . . . . . . . . 242

Review of Professor Peirce's Paper, "Pyorrhœa Alveolaris." By S. H. GUILFORD, D.D.S., Philadelphia . . . . . . . . . . 244

Review of Professor Peirce's Paper, "Pyorrhœa Alveolaris." By M. L. RHEIN, M.D., D.D.S., New York . . . . . . . . . . 247

Comments on Professor Peirce's Paper, "Pyorrhœa Alveolaris." By ALBERT P. BRUBAKER, M.D., D.D.S., Philadelphia . . . . . . . . . . 249

Comments on Professor Peirce's Paper, "Pyorrhœa Alveolaris." By EDWIN T. DARBY, M.D., D.D.S., Philadelphia . . . . . . . . . . 251

Report of an Operation. By DR. S. E. DAVENPORT, New York . . . . . 253

Combining Rubber Filings with Fresh Rubber. By DR. W. S. SIMONTON, West Virginia . . . . . . . . . . 255

REPORTS OF SOCIETY MEETINGS.

New York Odontological Society . . . . . . . . . . 256

American Academy of Dental Science . . . . . . . . . . 263

Odontological Society of Pennsylvania . . . . . . . . . . 270

EDITORIAL.

Medical Criticism . . . . . . . . . . 276

Pyorrhœa Alveolaris . . . . . . . . . . 281

Professor W. D. Miller . . . . . . . . . . 281

BIBLIOGRAPHY . . . . . . . . . . 282

NOTES AND COMMENTS . . . . . . . . . . 285

CURRENT NEWS.

Dental Society of the State of New York . . . . . . . . . . 285

Annual Meeting of Harvard Odontological Society . . . . . . . . . . 286

The Transactions of the World's Columbian Dental Congress . . . . . . 287

Central Dental Association of Northern New Jersey . . . . . . . . . . 287

SELECTIONS.

The Defect in the Dentist . . . . . . . . . . 288

